# Massive Delayed Cerebrospinal Fluid Leakage After Cervical Spinal Tumor Resection: A Case Report

**DOI:** 10.3390/jcm15145321

**Published:** 2026-07-08

**Authors:** In-Suk Bae, Hyoung-Joon Chun

**Affiliations:** 1 Department of Neurosurgery, Nowon Eulji Medical Center, Eulji University, Seoul 01830, Republic of Korea; tbgitw@eulji.ac.kr; 2 Department of Neurosurgery, Hanyang University Medical Center, Hanyang University, Seoul 04763, Republic of Korea

**Keywords:** cerebrospinal fluid leak, cervical spinal tumor, dural repair, muscle graft

## Abstract

**Background:** Cervical dumbbell-shaped neurogenic tumors occurring at two noncontiguous levels are rare, and postoperative cerebrospinal fluid (CSF) collection causing cord compression is an uncommon but serious complication after intradural tumor resection. **Case Presentation:** A 30-year-old man presented with a 3-month history of progressive gait disturbance. Neurological examination revealed grade 3 paraparesis with upper motor neuron signs. Magnetic resonance imaging (MRI) demonstrated two discrete dumbbell-shaped neurogenic tumors located at the C1-2 and C7-T1 levels. The lesions were simultaneously resected. Complete removal of the C1-2 tumor required total sacrifice of the left C2 nerve root, while the C7-T1 lesion was excised through a T-shaped dural incision. The dura was closed primarily with watertight sutures reinforced with dural sealant, and no CSF leakage was observed during intraoperative Valsalva testing. Two months postoperatively, the patient developed worsening upper back and trapezial pain with severe scapular swelling. MRI revealed a large CSF collection extending from C6 to T5, causing moderate cord compression. Urgent revision surgery was performed. Controlled drainage was attempted to prevent intracranial hypotension, but significant CSF egress occurred. The dural defect was repaired using an autologous muscle plug reinforced with fibrin glue. The patient recovered uneventfully after revision surgery and was discharged without recurrence or complications. **Conclusions:** This case highlights that delayed, extensive postoperative CSF collection can occur despite apparently watertight primary closure and negative intraoperative Valsalva testing. Clinical vigilance for this complication is essential when patients present with new axial pain or localized swelling following cervical intradural surgery, even in the absence of classic low-pressure headaches.

## 1. Introduction

Cerebrospinal fluid (CSF) leakage is a recognized complication of spine surgery that can lead to serious adverse outcomes such as wound infection, intracranial hemorrhage, nerve root strangulation, and meningitis [1,2,3,4,5,6,7,8,9]. Early recognition and appropriate management are essential to prevent these sequelae, particularly in intradural spinal tumor surgery, where the incidence of CSF leakage remains substantial despite watertight dural closure [10,11,12].

Persistent leakage increases the risk of secondary complications including meningitis, pseudomeningocele, and intracranial hypotension, often necessitating prolonged bed rest, lumbar CSF drainage, extended antibiotic therapy, or revision surgery [11,13,14,15]. Intracranial hypotension, driven by this volume depletion, classically presents as a debilitating orthostatic headache or rapid neurological decline. However, atypical presentations can easily escape early clinical suspicion.

We report a unique and clinically deceptive case of a delayed, massive postoperative CSF leak following resection of two cervical intradural dumbbell-shaped schwannomas. The purpose of this case report is to highlight that delayed CSF collection can occur despite apparently watertight primary closure and a negative intraoperative Valsalva test, to clarify the histologic diagnosis and operative outcome of the multifocal tumors, and to emphasize the need for clinical vigilance when new axial pain or localized swelling develops after cervical intradural surgery, even in the absence of classic low-pressure headache.

## 2. Case Description

### 2.1. Symptoms and Initial Presentation

A 30-year-old male presented to our clinic with a 3-month history of progressive gait disturbance and imbalance. He reported increasing difficulty with coordinated walking and mild, persistent discomfort in his upper neck region.

Upon thorough neurological examination, the patient demonstrated profound motor weakness, specifically grade 3 paraparesis (motor weakness in both lower extremities) with paresthesia (bilateral numbness and tingling in the hands and feet). Physical assessment further revealed upper motor neuron signs consistent with severe cervical myelopathy, including hyperactive deep tendon reflexes and bilateral positive pathologic reflexes (Babinski sign); sphincter function was completely preserved.

### 2.2. Preoperative Diagnosis

Magnetic resonance imaging (MRI) of the spine was performed to determine the etiology of the myelopathy. The sagittal and axial enhanced sequences demonstrated two separate, discrete, well-demarcated dumbbell-shaped neurogenic tumors on the left side (Figure 1). The superior lesion was situated at the C1-2 level, and the inferior lesion was located at the C7-T1 junction, both causing significant displacement and severe compression of the cervical spinal cord.

### 2.3. Primary Surgical Treatment and Interventions

Given the progressive neurological decline, simultaneous surgical resection of both intradural tumors was scheduled. The surgery was performed with concurrent intraoperative neuromonitoring (IONM), utilizing both transcranial motor evoked potentials (tcMEP) and somatosensory evoked potentials (SSEP). The patient was positioned concord position with neck flexion, and a posterior midline approach was used to expose the cervical C1-2 and C7-T1 levels.

C1-2 Lesion Resection: A posterior midline approach was used. The tumor was approached and removed intradurally. To ensure gross total removal of the extensive mass, the left C2 nerve root was completely sacrificed.

C7-T1 Lesion Resection: Because of the tumor’s extension into the neural foramen and nerve root sleeve, a standard linear durotomy was insufficient. A complex, T-shaped dural incision was performed over the spinal cord and the nerve root sleeve to achieve adequate exposure. A schematic illustration of the T-shaped dural incision is provided to clarify the operative geometry and the location of the later dural defect (Figure 2). Following gross total resection of the tumor, the T-shaped dural edges were meticulously reapposed.

Primary closure was executed using fine watertight sutures, which were subsequently reinforced with dural sealant (fibrin glue). An intraoperative Valsalva maneuver was performed up to standard physiological pressures; no active bubbling or CSF egress was observed, confirming an apparently robust, watertight seal.

Intraoperative neuromonitoring (IONM) remained stable throughout the procedure. Following drain placement, the operative wound was closed layer by layer.

Postoperative histopathological and immunohistochemical findings confirmed both lesions as schwannomas, with S100 positivity and EMA negativity, consistent with WHO classification of benign peripheral nerve sheath tumor. Because the patient had two anatomically noncontiguous nerve sheath tumors, we reviewed the possibility of an underlying tumor-predisposition syndrome. Family history was negative for neurofibromatosis, schwannomatosis, or other related hereditary disorders.

### 2.4. Postoperative Course and Delayed Presentation

Directly following the primary operation, the patient showed excellent neurological recovery, with lower extremity motor strength improving significantly from grade 3 to grade 4. He reported mild residual periscapular pain, but his surgical wounds healed cleanly without any localized fluid collections, and he was safely discharged home.

However, two months postoperatively, the patient returned with a severe exacerbation of pain localized to the upper back and bilateral trapezius muscles. Physical examination revealed tense localized swelling across the scapular and paraspinal area. There was no accompanying deterioration in motor function, and he did not report an orthostatic headache or other classic symptoms of intracranial hypotension.

### 2.5. Secondary Diagnosis and Urgent Intervention

An urgent follow-up MRI was ordered to evaluate the expanding paraspinal mass. The imaging revealed a massive pseudomeningocele (CSF collection) tracking extensively along the posterior elements from C6 down to T5, which was exerting moderate mass effect and direct compression on the spinal cord (Figure 3). A separate, substantially smaller fluid collection was noted superiorly at the C1-2 operative site.

The patient was taken immediately to the operating room for urgent revision surgery. To mitigate the risk of acute intracranial hypotension from rapid pressure decompression, the fascia was opened via a small initial track. Despite this controlled attempt, a substantial and forceful egress of trapped CSF occurred upon entering the cavity. Meticulous microdissection was performed down to the previous durotomy sites. The C1-2 site was intact, but a distinct 2 mm dural defect was discovered precisely at the triradiate junction of the previous T-shaped incision at the C7-T1 level.

To permanently close this high-stress geometric point, the dural defect was plugged using an autologous muscle graft harvested from the local paracervical musculature. This muscle plug was securely layered and reinforced with a generous application of fibrin glue. Watertight fascial and cutaneous closures were then performed.

### 2.6. Treatment Outcomes

Following the revision surgery, the patient’s recovery was smooth and entirely uneventful. His severe trapezial and axial back pain resolved rapidly as the local mass effect subsided. The subcutaneous paraspinal swelling completely disappeared. At his final follow-up on postoperative 10 months, he remained neurologically stable with no signs of pseudomeningocele recurrence, wound complications, or delayed CSF leaks (Figure 4).

## 3. Discussion

This case highlights delayed massive CSF leakage after cervical intradural dumbbell tumor surgery without the classic symptoms of intracranial hypotension. The leakage site was successfully repaired with an autologous muscle graft, leading to a favorable outcome.

CSF leakage during spine surgery occurs in approximately 0.2–20% of cases, but the reported range varies widely across studies because procedures, pathology, and definitions differ; selected series of intradural tumor surgery report higher rates than routine degenerative procedures [16,17]. Reported risk factors for CSF leakage after spinal cord tumor resection include reoperation, extensive laminectomy, and revision surgery [18]. Despite advances in surgical technique, the persistence of CSF leakage underscores a significant complication burden and justifies further investigation, especially in intradural spinal tumor surgery where intentional dural opening is mandatory.

Dumbbell-shaped intradural tumors of the cervical spinal canal represent a complex subset of spinal tumors. They are technically demanding and hazardous to manage compared to simple benign tumors, carrying higher rates of residual disease and local recurrence. Tumor extension into the nerve root sleeve often requires nonstandard, complex dural incision patterns, which may increase the risk of structural closure failure. After the resection of a large dumbbell tumor, the resulting dural defect can be extensive, potentially leading to substantial CSF loss, intracranial hypotension, and consequent changes in intracranial compartment volumes.

In our patient, the dural defect was located at the junction of the prior T-shaped incision at the C7-T1 level. This observation suggests a possible, but unproven, relationship between complex dural geometry and delayed closure failure, particularly in a deep operative field where the dural edges may be under tension. We therefore interpret the junctional defect as a plausible site of stress concentration rather than as definitive evidence of intrinsic structural vulnerability. Additional reinforcement, such as an autologous muscle graft or patch material, may be considered when the initial dural incision is complex or the defect is large.

Ideally, the recognition of a dural breach should occur intraoperatively. Unintended durotomies may be signaled by CSF escaping into the surgical field, a sudden increase in epidural venous bleeding, or a decrease in thecal sac turgor. In intended durotomies, primary closure failure is typically elicited intraoperatively using a Valsalva maneuver. However, as this case demonstrates, a negative intraoperative Valsalva test does not entirely rule out delayed dural dehiscence under postoperative physiological pressure.

Persistent CSF leaks can cause numerous complications, including delayed wound healing, meningitis, pseudomeningocele formation, and low-pressure sequelae like cerebellar herniation or cranial nerve deficits [4,7,19]. Intracranial hypotension typically manifests as a debilitating orthostatic headache that worsens when upright and improves when flat, caused by CSF hypovolemia and the subsequent downward displacement of brain structures.

In our case, the patient lacked an orthostatic headache despite a massive fluid collection. One possible explanation is that the dural defect was very small, allowing for gradual CSF egress over a prolonged period and thereby limiting abrupt intracranial pressure shifts. This is a hypothesis only and cannot be proven from this case alone. The absence of a classic headache may therefore be misleading and should not exclude delayed CSF leakage in patients who present with new axial pain or localized swelling after intradural surgery.

Consequently, clinicians should maintain a high index of suspicion for delayed leaks in any patient who presents with new localized swelling or axial pain after intradural operations, regardless of headache absence. When complex incisions are used initially, primary reinforcement with autologous tissue plugs or patch matrices may be considered to reduce the risk of postoperative leakage; however, this suggestion is based on a single case and should not be interpreted as a general rule.

This case report has several limitations. First, it describes a single patient, so the findings cannot be generalized. Second, the proposed mechanism of delayed dural failure at the T-shaped incision site is inferential and cannot be proven definitively. Third, because no comparative cohort or alternative closure strategy was available, the report cannot determine the relative superiority of any particular dural repair method. Therefore, the present findings should be interpreted as a clinical observation rather than definitive evidence.

Future work could use parametric finite element models to evaluate how different dural incision geometries and suture configurations influence local stress distribution at the closure line. Such modeling could be paired with experimental validation and, in the longer term, machine-learning-based predictive frameworks to support rapid optimization of dural closure strategies.

## 4. Conclusions

Delayed CSF leakage should be strongly suspected in patients who develop new axial pain or localized swelling after cervical intradural tumor surgery, even in the absence of an orthostatic headache. This single case suggests that complex, non-linear dural incisions such as T-shaped cuts may be vulnerable to delayed failure and may represent sites of stress concentration; therefore, primary reinforcement with autologous tissue grafts may be considered during initial closure. When delayed accumulation occurs, prompt surgical revision incorporating durable dural repair with an autologous muscle plug and fibrin glue can provide excellent clinical outcomes.

## Figures and Tables

**Figure 1:**
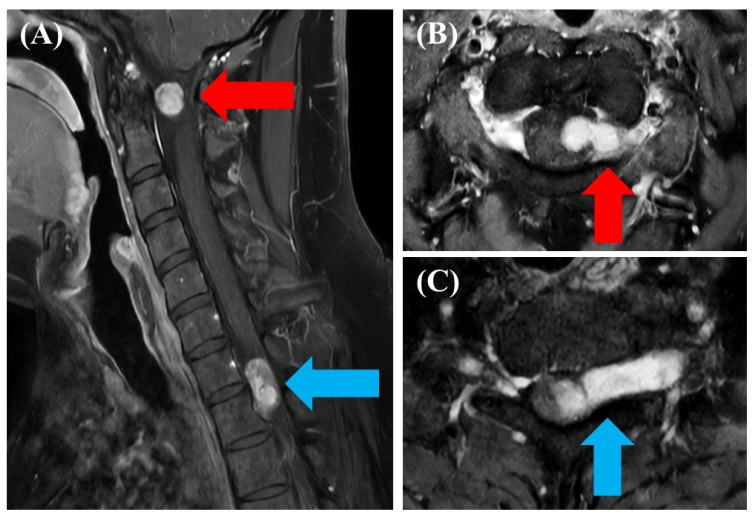
Dumbbell-shaped neurogenic tumors located at the C1-2 and C7-T1 levels. (**A**) Sagittal enhanced image showing C1-2- and C7-T1-level neurogenic tumors. (**B**) Axial image of the C1-2-level tumor (Red arrow). (**C**) Axial image of the C7-T1-level tumor (Blue arrow).

**Figure 2:**
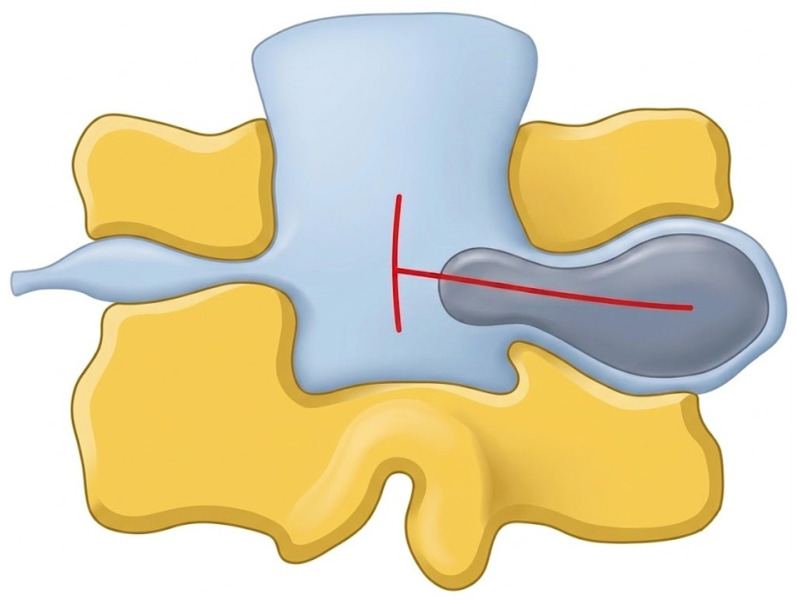
Schematic illustration of the T-shaped dural incision at the C7-T1 level.

**Figure 3:**
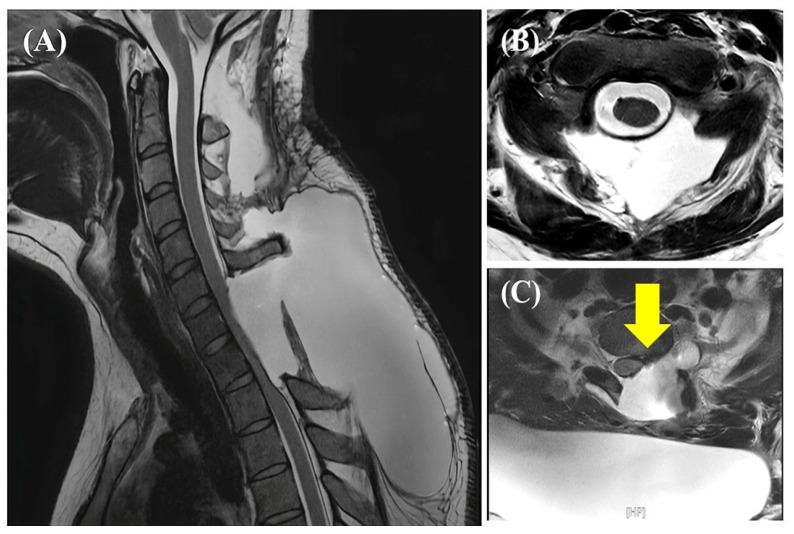
Massive CSF collection extending from C6 to T5. (**A**) Sagittal image showing the massive CSF collection. (**B**) Axial image of the C1-2-level CSF collection. (**C**) Axial image of the C7-T1-level CSF collection (Yellow arrow showing the dural defect site at the triradiate junction of the previous T-shaped incision at the C7-T1 level).

**Figure 4:**
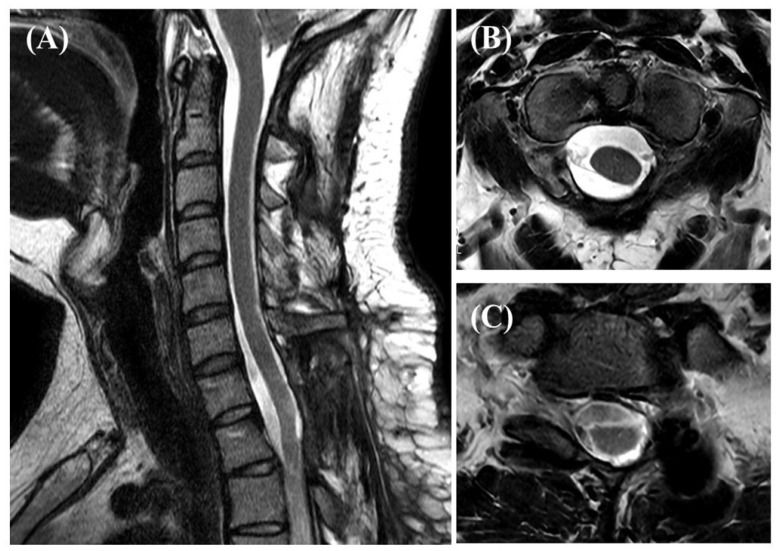
Final follow-up MRI on postoperative 10 months showing no signs of pseudomeningocele recurrence, or delayed CSF leaks. (**A**) Sagittal image showing no CSF leakage. (**B**) Axial image of the C1-2 level. (**C**) Axial image of the C7-T1 level.

## Data Availability

The data presented in this study are available upon request from the corresponding author. No new data were created or analyzed in this study.

## Abbreviations

The following abbreviations are used in this manuscript:

CSF	Cerebrospinal fluid
MRI	Magnetic resonance imaging
